# Chemical Immobilization of Sloth Bears (*Melursus ursinus*) with Ketamine Hydrochloride and Xylazine Hydrochloride: Hematology and Serum Biochemical Values

**DOI:** 10.1155/2014/341047

**Published:** 2014-04-28

**Authors:** M. Veeraselvam, R. Sridhar, P. Perumal, M. G. Jayathangaraj

**Affiliations:** ^1^Department of Wild Life Science, Madras Veterinary College, Tamil Nadu Veterinary and Animal Sciences University, Chennai 600 007, India; ^2^Veterinary University Training and Research Centre, Tamil Nadu Veterinary and Animal Sciences University (TANUVAS), Salem 636 001, India; ^3^Department of Veterinary Pathology, Madras Veterinary College, TANUVAS, Chennai 600 007, India; ^4^National Research Centre on Mithun, ICAR, Jharnapani, Nagaland 797106, India

## Abstract

The present study was conducted to define the physiological responses of captive sloth bears immobilized with ketamine hydrochloride and xylazine hydrochloride and to determine and compare the values of hematology and serum biochemical parameters between sexes. A total of 15 sloth bears were immobilized using combination of ketamine hydrochloride and xylazine hydrochloride drugs at the dose rate of 5.0 milligram (mg) per kg body weight and 2.0 mg per kg body weight, respectively. The use of combination of these drugs was found satisfactory for the chemical immobilization of captive sloth bears. There were no significant differences observed in induction time and recovery time and physiological parameters such as heart rate, respiratory rate, and rectal temperature between sexes. Health related parameters comprising hematological values like packed cell volume (PCV), hemoglobin (Hb), red blood cell count (RBC), erythrocyte indices, and so forth and biochemical values like total protein, blood urea nitrogen (BUN), creatinine, alkaline amino-transferase (ALT), aspartate amino-transferase (AST), and so forth were estimated in 11 (5 males and 6 females) apparently healthy bears. Comparison between sexes revealed significant difference in PCV (*P* < 0.05) and mean corpuscular hemoglobin concentration (MCHC) (*P* < 0.05). The study might help to evaluate health profiles of sloth bears for appropriate line treatment.

## 1. Introduction

Chemical immobilization is considered a necessary component for research and management purposes of wild animals [1–3]. The combinations of anesthetics like ketamine and xylazine have been widely used for domestic and nondomestic animals [2, 4, 5]. This combination has also been used for Ursids [6–8]. It has been observed that when ketamine hydrochloride has been used alone as an immobilizing or anesthetic agent, it has led to muscle rigidity and convulsions in animals [3, 9]. Hence, ketamine hydrochloride is mixed with xylazine hydrochloride, which acts as a muscle relaxant and helps reduce incidence of muscle convulsions in the animal and makes it less rigid during handling [6, 7, 10]. The combination of these two drugs, namely, xylazine hydrochloride and ketamine hydrochloride, has been found to generally result in smooth induction and recovery of the animal [11]. The results mentioned in the present study have been obtained after combination of ketamine hydrochloride and xylazine hydrochloride was used to immobilize sloth bears (*Melursus ursinus*) for general health checkup at the Nehru Zoological Park, Hyderabad.

In the present study, we have been able to obtain the mean hematological and serum biochemical values for Indian sloth bears, which will prove to be a useful data to evaluate health profiles of sloth bears. Research on health and diseases on captive bear species and availability of related information on the same is still in a state of infancy in India. Understanding of the health related parameters and evidence of diseases in captive-reared bears will thus significantly contribute towards enriching the management of captive bears in India. Blood constituents can be used to monitor the health status and nutritional deficiencies and diagnose diseases of animals. In this regard, establishing a standard baseline data of hematological and serum biochemical values for captive sloth bears will prove to be beneficial not only for research and monitoring purposes but also for the medical treatment purposes of sloth bears [12].

## 2. Materials and Methods

The present study was performed at the Bear Rescue Transit Facility, located within Nehru Zoological Park, Hyderabad, Andhra Pradesh, India (17.366°N; 78.470E) during the period from June 2006 to April 2007. A total of 15 apparently healthy-looking sloth bears included 7 males and 8 females with varying age groups ranging from 2 years to 16 years were used for purpose of this study. The bears were maintained in separate enclosure with the same age groups. The diets of bears were grains gruel and local seasonal fruits.

Prior to chemical immobilization, the sloth bear to be immobilized was kept isolated in a separate enclosure and was fasted overnight though water was made available to the bear. Blowpipe was used for darting the animal. Prior to injection, the dosage of the immobilizing drugs used was calculated for the bear based on their body weight as per the following dose rate, that is, 5 mg of ketamine hydrochloride (Ketamil, Troy laboratories Pty Ltd., Smithfield, NSW, Australia) per kg of body weight and 2 mg of xylazine hydrochloride (Xylazil, Troy laboratories Pty Ltd., Smithfield, NSW, Australia) per kg of body weight. The sloth bear was darted around the shoulder or neck region, since these areas have thinner deposition of subcutaneous fats, which thereby increases the probability of the immobilizing drugs penetrating efficiently into the highly muscular area of the animal [6]. After anesthetic induction of the animal, the eyes of the bear were blind folded with a piece of cloth to avoid corneal damage. Ambient temperature during chemical immobilization of the sloth bears ranged between 29.5°C and 31.1°C.

Induction time was recorded as the time taken by the bear from being injected till the time at which the animal attained sternal or lateral recumbency. Respiration rate, resting heart rate, and rectal temperature were recorded after the animal attained full lateral recumbency. Recovery time was calculated as the time interval between recumbency and the animals ability to maintain standing posture.

Whole blood and serum were collected from each of the 15 sloth bears to assess their standard health related parameters. Blood was collected from the jugular vein of the animal using 18-gauge sterile hypodermic needle in vacutainer tubes containing an anticoagulant (Vacutainer, Becton Dickinson Vacutainer Systems, Franklin Lakes, NJ, USA) for hematological examination and in plain vacutainer tubes for serum biochemical studies. Blood smears from each animal were prepared for differential count. Serum was separated by centrifugation at 2000 rpm for 10 min and the serum was stored at −20°C until further processing.

The erythrocyte sedimentation rate (ESR) was measured using Westegreen pipette. The packed cell volume (PCV) was estimated by the microhaematocrit method. The haemoglobin (Hb) was measured by acid-haematic method using a haemoglobinometer. The red blood cell count (RBC) and white blood cell count (WBC) were done using a haemocytometer. The determined values were then used to calculate the erythrocytic indices, namely, the mean corpuscular volume (MCV), mean corpuscular haemoglobin (MCH), and mean corpuscular haemoglobin concentration (MCHC). The haematological examination was carried out as per the methods described by Schalm et al. [13].

Serum biochemical values including organ function profiles such as uric acid, blood urea nitrogen (BUN), creatinine, and total bilirubin, blood metabolic profile such as total protein, albumin, globulin, blood glucose, serum cholesterol, calcium, and phosphorus, and serum enzyme profile such as alanine amino-transferase (ALT), aspartate amino-transferase (AST), and alkaline phosphatase (ALP) were obtained using span diagnostic test kit with semiautoanalyzer (CECIL instruments limited, Cambridge, England) with standard techniques.

Basic statistics including mean, median, and standard deviation were determined for each variable. For normally distributed variables, a 95% confidence interval for means was calculated. The statistical analysis of data was carried out using sample *t*-test, as per the standard procedures given by Snedecor and Cochran [14].

## 3. Results

A total of 15 sloth bears were immobilized using combination of ketamine hydrochloride and xylazine hydrochloride drugs. The dose for ketamine hydrochloride and xylazine hydrochloride was calculated at 5.0 mg per kg body weight and 2.0 mg per kg body weight, respectively. After darting, early signs of drug effects such as mydriasis and ataxia were observed before the animal slumps into sternal recumbency. Salivation was observed in most of the bears, but emesis was not observed because food was withheld from the animal for at least 12 hours prior to their chemical immobilization. The induction time ranged from 9 to 21 minutes (mean—12.04 min; median—11; SE—2.86; *n*—15). Finally muscles of the neck and head were completely relaxed. The recovery time ranged from 50 to 175 minutes (mean—125.2; SE —50.73; median—135; *n*—15). There were no significant differences in the induction time and recovery time between sexes.

Heart rate, respiratory rate, and rectal temperature were measured after the animal went into complete recumbency. Heart rates ranged from 66 to 84 beats per minute (mean—73.4; SE —5.59; median—72; *n*—15). The range of respiratory rate was from 12 to 28 per minute (mean—16.8; SE —4.53; median—16; *n*—15) and the rectal temperature range was 36.2°C to 39.9°C (mean—38.2°C; SE —0.92; median—38.2°C; *n*—15) per minute. There were no significant differences observed in heart rate, respiratory rate, and rectal temperature between sexes.

Early stages of recovery of the animal were marked with blinking of eyes, twitching of the ears, and response to external stimuli. The bear's initial effort to stand on its leg showed a walk in uncoordinated movement and slowness. They, however, responded to tactile and auditory stimuli and were not aggressive.

A total of 11 samples from apparently healthy bears (5 males and 6 females) were subjected to hematological and serum biochemical analysis. Comparisons were also made between males and females. The overall mean (±SE) values obtained for hematological and serum biochemical parameters are given in Tables 1 and 2. Comparison of the values obtained on the basis of the sex determined that the female sloth bear had a significantly (*P* < 0.05) higher mean level of PCV than males. Additionally, males had a significantly (*P* < 0.05) higher mean level of MCHC than females. There were no significant differences observed in other hematological and serum biochemical parameters.

## 4. Discussion

Ketamine hydrochloride and xylazine hydrochloride have received widespread use as chemical immobilizing drugs for nondomestic carnivores [15]. In the present study the use of combination of these drugs was found satisfactory for the chemical immobilization of captive sloth bears. The approximate dose of 5 mg of ketamine hydrochloride per kg of body weight and 2 mg of xylazine hydrochloride per kg of body weight produced adequate immobilization of sloth bears for the purpose of health checkup and sample collection. These doses were lower than the values used by other investigators working with this combination in sloth bears [7, 16]. The advantages of inducing chemical immobilization with a combination of ketamine hydrochloride and xylazine hydrochloride have been well documented [6, 9, 10] and the visible advantages of using this combination have been confirmed by this study, as has been characterized by rapid inductions, smooth recoveries, and adequate muscle relaxation for most procedures. The wide safety margin of these drugs permits additional injection of drug whenever necessary [2].

The mean induction time as observed in our study is slightly longer than reported by other authors in sloth bears [7, 16]. The need in some of our cases for a second dosage of anesthetics may be related to the targeted injection site and in some cases it may be due to the fact that the injection would have remained subcutaneous and the constituents in the dart would not have entered the muscle mass. Drugs injected subcutaneously or into fat deposits resulted in reduced degree of immobilization and a prolonged recovery time due to poor absorption [10]. In this study, it was observed that dosage levels of ketamine hydrochloride and xylazine hydrochloride produced no significant differences in respiration rate, pulse rate, and body temperature as measured immediately after the animals went to full recumbency. The overall mean of these values obtained in this study was in agreement with the values given by Page [7].

The hematology and serum biochemistry data obtained in this study showed minor but insignificant differences in some values reported by Bush et al. [17] and Wallach and Boever [18]. However, when the parameters obtained were compared between sexes, there was significant increase noted in the PCV values in females when compared to those in males. This might be attributed due to haemoconcentration and cyclic breeding stages of the animal. The MCHC values obtained for male sloth bears in this study had a significant difference when compared to the females. Shanmugam et al. [19] also reported significant differences in MCV, MCH, and MCHC values between sex and geographical locations in sloth bears. This was in agreement with Matula et al. [20] and the results cited in Schroeder [21] who attributed that the decrease might be due to iron deficiency (anemia) in females. Moreover, minor differences were observed when compared to other studies in both hematology and serum biochemistry parameters. These may require further standardization of collection, analytical techniques, and studies to understand geographical variations that may exist in both wild and captive sloth bears residing in different regions.

## Figures and Tables

**Table 1:** Hematology values for sloth bears (sex comparison and combined value).

Parameters	Male (*n* = 5) (mean ± SE)	Female (*n* = 6) (mean ± SE)	Sex combined (mean ± SE)	95% confidence interval
PCV (%)	49.80^a^ ± 0.86	53.83^b^ ± 1.40	52 ± 1.03	51.4–52.6
Haemoglobin (g/dL)	15.72 ± 1.09	17.92 ± 0.47	16.92 ± 0.63	16.55–17.29
Erythrocyte (10^6^/mm^3^)	6.53 ± 0.25	6.59 ± 0.31	6.56 ± 0.19	6.45–6.67
ESR (mm per 60 min)	5.60 ± 0.92	6.33 ± 0.33	6.0 ± 0.45	5.73–6.27
MCV (fL)	76.64 ± 2.97	82.43 ± 3.14	79.79 ± 2.26	78.45–81.13
MCH (pg)	25.75 ± 1.05	27.40 ± 1.05	26.65 ± 0.75	26.21–27.09
MCHC (%)	33.57^a^ ± 0.11	33.28^b^ ± 0.05	33.41 ± 0.07	33.37–33.45
Leukocyte (10^3^/mm^3^)	12.14 ± 0.63	11.37 ± 0.26	11.72 ± 0.32	11.53–11.91
Lymphocytes (10^3^/mm^3^)	2.82 ± 0.13	2.88 ± 0.17	2.85 ± 0.10	2.79–2.91
Neutrophils (10^3^/mm^3^)	8.54 ± 0.33	7.99 ± 0.20	8.24 ± 0.19	8.13–8.35
Monocytes (10^3^/mm^3^)	0.45 ± 0.13	0.28 ± 0.04	0.36 ± 0.06	0.32–0.4
Eosinophils (10^3^/mm^3^)	0.25 ± 0.09	0.16 ± 0.02	0.20 ± 0.44	[−0.06–0.46]
Basophils (10^3^/mm^3^)	0.08 ± 0.03	0.07 ± 0.04	0.07 ± 0.02	0.06–0.08

^a,b^Statistically significant from the opposite sex (*P* ≤ 0.05).

**Table 2:** Serum biochemical values for sloth bears (sex comparison and combined value).

Parameters	Male (*n* = 5) (mean ± SE)	Female (*n* = 6) (mean ± SE)	Sex combined (mean ± SE)	95% confidence interval
Calcium (mg/dL)	9.84 ± 0.49	9.16 ± 0.33	9.47 ± 0.29	9.3–9.64
Phosphorus (mg/dL)	5.29 ± 0.18	4.96 ± 0.24	5.11 ± 0.15	5.02–5.20
Uric acid (mg/dL)	3.70 ± 0.29	3.95 ± 0.22	3.84 ± 0.17	3.74–3.94
Total protein (g/dL)	6.57 ± 0.41	7.17 ± 0.45	6.98 ± 0.30	6.8–7.16
Albumin (g/dL)	3.64 ± 0.19	4.11 ± 0.29	3.89 ± 0.19	3.78–4
Globulin (g/dL)	3.11 ± 0.26	3.07 ± 0.21	3.09 ± 0.15	3–3.18
A/G Ratio	1.19 ± 0.08	1.35 ± 0.10	1.27 ± 0.07	1.23–1.31
Glucose (mg/dL)	101.40 ± 2.38	96.67 ± 2.88	98.82 ± 1.96	97.66–99.98
Total cholesterol (mg/dL)	276.20 ± 8.13	262.33 ± 6.32	268.63 ± 5.22	265.55–271.71
BUN (mg/dL)	20.02 ± 0.86	18.30 ± 0.58	19.08 ± 0.55	18.75–19.41
Creatinine (mg/dL)	1.10 ± 0.14	1.35 ± 0.12	1.24 ± 0.09	1.19–1.29
Total bilirubin (mg/dL)	0.18 ± 0.04	0.13 ± 0.07	0.15 ± 0.04	0.13–0.17
ALP (IU/L)	35.10 ± 2.60	37.51 ± 2.04	36.41 ± 1.58	35.48–37.34
AST (IU/L)	133.42 ± 3.02	141.33 ± 4.89	137.74 ± 3.11	135.9–139.58
ALT (IU/L)	104.84 ± 3.01	102.50 ± 2.61	103.56 ± 1.90	102.44–104.68
